# A Wearable Diagnostic Assessment System vs. SNAP-IV for the auxiliary diagnosis of ADHD: a diagnostic test

**DOI:** 10.1186/s12888-022-04038-3

**Published:** 2022-06-21

**Authors:** Jie Luo, Huanhuan Huang, Shuang Wang, Shengjian Yin, Sijian Chen, Lin Guan, Xinlong Jiang, Fan He, Yi Zheng

**Affiliations:** 1 The National Clinical Research Center for Mental Disorders & Beijing Key Laboratory of Mental Disorders, Beijing Anding Hospital & the Advanced Innovation Center for Human Brain Protection, Capital Medical University, Beijing, China; 2 China Medical University, Shenyang, China; 3 Institute of Computing Technology, CAS, Beijing, China

**Keywords:** Attention-deficit/hyperactivity disorder (ADHD), Diagnosis, Wearable computing, Objective

## Abstract

**Objective:**

We design a diagnostic test to evaluate the effectiveness and accuracy of A non-intrusive Wearable Diagnostic Assessment System versus SNAP-IV for auxiliary diagnosis of children with ADHD.

**Methods:**

This study included 55 children aged 6–16 years who were clinically diagnosed with ADHD by DSM-5, and 55 healthy children (typically developing). Each subject completes 10 tasks on the WeDA system (Wearable Diagnostic Assessment System) and Parents of each subject complete the SNAP-IV scale. We will calculate the validity indexes, including sensitivity, specificity, Youden's index, likelihood ratio, and other indexes including predictive value, diagnostic odds ratio, diagnostic accuracy and area under the curve [AUC] to assess the effectiveness of the WeDA system as well as the SNAP-IV.

**Results:**

The sensitivity (94.55% vs. 76.36%) and the specificity (98.18% vs. 80.36%) of the WeDA system were significantly higher than the SNAP-IV. The AUC of the WeDA system (0.964) was higher than the SNAP-IV (0.907). There is non-statistically significant difference between groups (*p* = 0.068), and both of them have high diagnostic accuracy. In addition, the diagnostic efficacy of the WeDA system was higher than that of SNAP-IV in terms of the Youden index, diagnostic accuracy, likelihood ratio, diagnostic odds ratio and predictive value.

**Conclusion:**

The advantages of the WeDA system in terms of diagnostic objectivity, scientific design and ease of operation make it a promising system for widespread use in clinical practice.

## Keypoints

- Prior to this study, there were no diagnostic trial studies of wearable devices to auxiliary diagnosis of ADHD.
- Compared to previous wearable devices systems, the self-developed WeDA system is based on the DSM-5 diagnostic criteria and is scientifically designed with a high degree of conviction.
- The results of the study showed that the WeDA system had a sensitivity of 94.55%, a specificity of 98.18% and a diagnostic accuracy of 96.36%, much higher than the rating scale SNAP-IV.
- These results demonstrate that the WeDA system has good auxiliary diagnostic value and has great potential for widespread clinical use.

## Introduction

Attention-deficit/hyperactivity disorder (ADHD) is one of the most common neuropsychiatric disorders during childhood and adolescence. The worldwide prevalence of ADHD in children is estimated to be 7.2% [1]. It is characterized by inattention, hyperactivity and impulsivity, accompanied by cognitive, emotional and behavioral impairments [2]. In the absence of diagnosis and treatment, some patients’ symptoms may continue from childhood into adulthood [3]. These symptoms make the person dysfunctional at home, in social intercourse and at work. Therefore, early diagnosis and intervention of ADHD is particularly important.

According to the *Chinese Guidelines for the Prevention and Treatment of Attention Deficit Hyperactivity Disorder* edited by Zheng et al. [4]*,* ADHD currently lacks etiological or pathological changes of diagnostic significance, and there are few objective signs and laboratory tests to assist in the diagnosis. For a diagnosis of ADHD, patient’s symptoms should meet the DSM-5 (the Diagnostic and Statistical Manual of Mental Disorders, 5th ed)/ICD-10 (International Classification of Diseases, tenth Revision) diagnostic criteria. However, during the clinical interview, children with ADHD are often not clear in expressing themselves and are uncooperative, while parents or teachers are often subjective in their descriptions of the child's symptoms. The diagnosis of ADHD is a complex and subjective process. In addition, there are very few experts in China that can diagnose ADHD and the demand for its diagnosis is far from being met [5].

The provision of reliable diagnostic aids for clinicians can greatly improve diagnostic efficiency. Currently, the most commonly used diagnostic aids in clinical practice are rating scales [6]. Several rating scales have been developed to measure ADHD according to the DSM. The Swanson, Nolan, and Pelham scale version IV (SNAP-IV) [7], one of the most widely used questionnaires in clinical practice, is a behavioural scale that measures the core symptoms of ADHD and oppositional defiant disorder as defined by the DSM-IV (American Psychiatric Association, 2013) and can be completed by teachers and parents. The validity of SNAP-IV was confirmed in many studies around the world, including the United States [8], China [9], Japan [10], Brazil [11], and Argentina [12]. However, measurement invariance was its greatest weakness. Hall CL et al. [13] found no measurement invariance between parents and teachers. The study by Lúcio PS et al. [14] with pre-school children also validated this result and indicated that there was approximate measurement invariance for teachers' assessments over a longitudinal interval of 6 months. More specifically, recent study suggested that girls were more predictable than boys in terms of ADHD according to both parents and teachers [15]. Other scales may also suffer from the above problems. These scales are generally descriptive questionnaires, lacking quantitative criteria, and easily influenced by the subjective experience.

Nowadays, objective diagnostic ADHD methods based on wearable inertial sensors (e.g. accelerometers and gyroscopes) have been studied [16, 17]. They are relatively inexpensive and easy to operate without requirement for additional monitoring. The low power consumption sensors like accelerometers can record the user's motion-related signals for long periods of time without disturbing their daily activities. More importantly, they measure the most realistic motion feature of the user’s daily life, thus increasing ecological validity [18].

At present, using wearable inertial measurement units, the Institute of Computing Technology, Chinese Academy of Sciences and Beijing Anding Hospital co-developed a new Wearable Diagnostic Assessment System based on the clinical diagnostic criteria of DSM-5 and six classical detection paradigms and four interaction modes. In this study, we aimed to evaluate the accuracy and validity of the WeDA system in the diagnosis of ADHD and compare it with the SNAP-IV scale.

## Methods

### Study design

This study followed the STARD statement (Standards for Reporting of Diagnostic Accuracy Studies [19]. The area under the receiver operating curve (ROC) of previous similar wearable devices was 0.9 or more at α = 0.05 (unilateral), β = 0.1, and a 1:1 ratio between groups [20, 21]. The sample size was estimated using PASS15 software (NCSS LLC.,Kaysville, U.T., USA) and a minimum of 55 participants with ADHD and 55 controls were needed after considering a 10% of lost to follow-up. All subjects were tested on the WeDA system, with the SNAP-IV scale being completed by the subject's parents.

### Subjects

Subjects in ADHD group were outpatients of Beijing Anding Hospital. The inclusion criteria were as follows: (a) meeting the DSM-5 diagnostic criteria for attention deficit and hyperactivity disorder; (b) aged 6–16 years and normal corrected visual acuity regardless of gender; (c) Wechsler Intelligence Scale for Children, Fourth Edition (WISC-CR) IQ ≥ 70; (d) being cooperative with wearing and completing games; (e) no previous use of medication for ADHD; (f) informed consent were obtained from patient and guardian. The exclusion criteria were as follows: (a) severe physical illness, closed-angle glaucoma, and previous history of epilepsy; (b) comorbidity with various developmental disorders such as mental retardation and autism spectrum disorder; (c) comorbid severe psychiatric disorders such as schizophrenia and bipolar disorder; (d) other conditions that the investigator considers unsuitable for inclusion in the group. The control group recruited children with normal development and excluded other disorders. In addition, children with symptoms of ADHD scored by the SNAP-IV while did not meet the diagnosis of ADHD under the gold standard were also included in the control group. All children were able to complete all the requirements of the trial. The project was approved by the Ethics Committee of Beijing Anding Hospital (201743FS-2).Finally, 110 subjects were successfully recruited and none of them dropped out. All subjects signed an informed consent form before the trial. All subjects completed the SNAP-IV scale and the WeDA system test, and the data were reliable and valid.

### Gold standard

The gold standard for the diagnosis of ADHD was based on the the opinion of two senior experts (Qi Yanjie and He Fan). According to the DSM-5 diagnostic criteria, all subject had significant symptoms of ADHD that significantly affecting their learning and life after ruling out other disorders [22]. Reliable information about the diagnosis was obtained from a detailed clinic interview between the two senior specialists and the subject’s family, as well as from clinical observations of the subject, combined with certain physical examinations to rule out other causes of the symptoms. Finally, the unanimous opinion of two senior experts was deemed as the gold standard for diagnosis [23].

### WeDA system and SNAP-IV

**Fig. 1:**
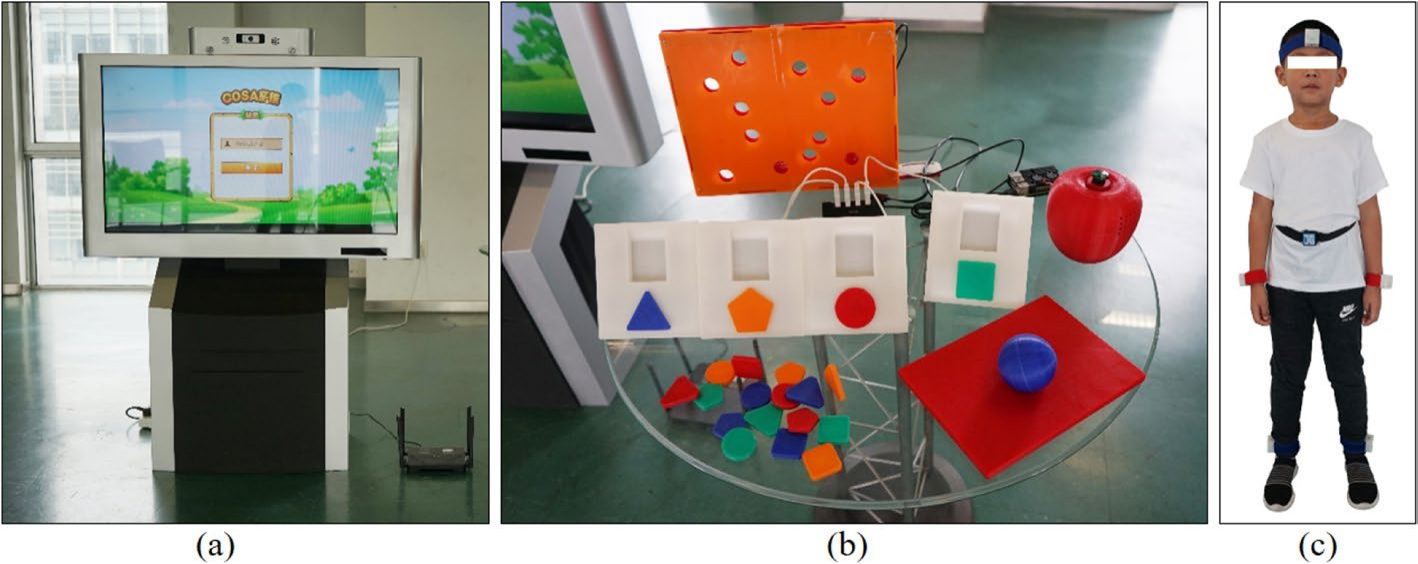
WeDA system hardware part (**a**) A big touch-screen (**b**) some 3D printing physical devices, and (**c**) Motion sensors

**Fig. 2:**
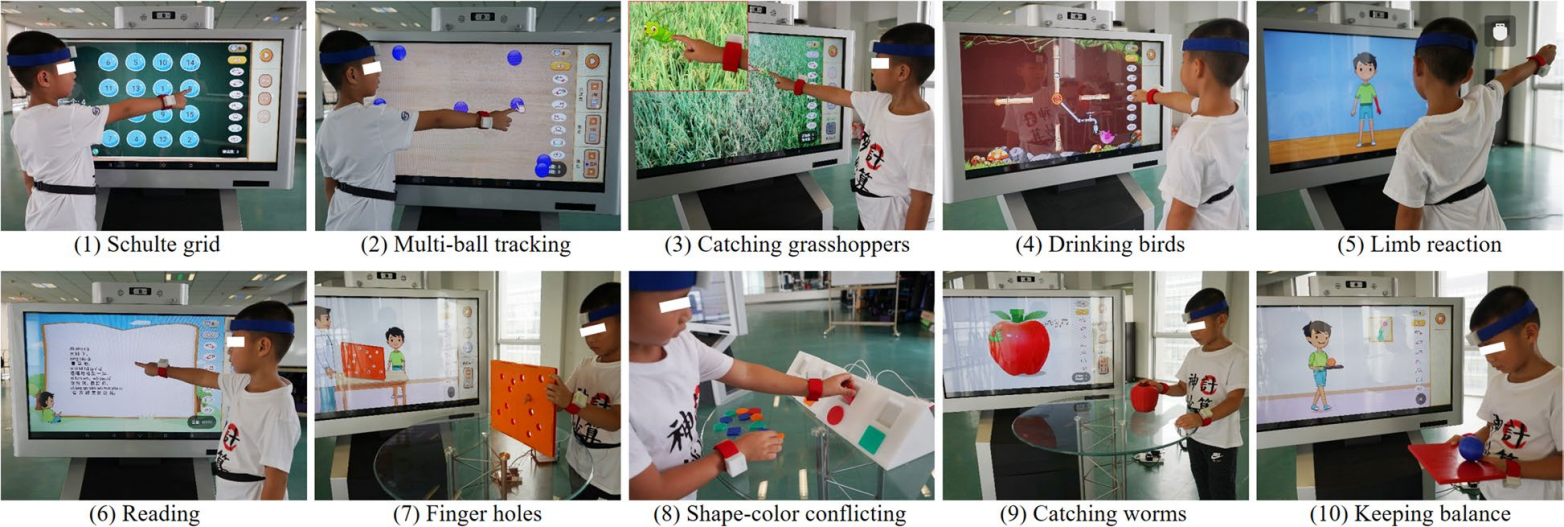
Different game tasks in WeDA system

The WeDA system is a wearable device diagnostic system based on the DSM-5 and designed to combine a variety of classical psychological paradigms. The system consists of a computer machine with a large touch screen, a set of 3D printed interactive devices, and six wearable motion sensors (Fig. 1). It included ten tasks, including: schulte grid, multi-ball tracking, catching grasshoppers, drinking birds, limb reaction, reading, finger holes, shape-color conflicting, catching worms, and keeping balance (Fig. 2). The user is first asked to wear six motion sensors on their head, hands, feet and waist. Then they completed ten tasks by touching the screen or interacting with the 3D printed device within a set time frame. During this process, students’ performance is scored not only based on the completion of the tasks (including accuracy, error rate, time consumption and other information) but also on the user’s body posture (obtained through the wearable device). User’s body movements are observed via six motion sensors. By integrating this information, Random forest and Bayesian network were employed to build diagnosis models. Details about the WeDA system have been published by our research team [24].

The parent version of the SNAP-IV was used to evaluate children with ADHD as it was closer to the clinical diagnosis [25]. The 26 items in the parent version of the SNAP-IV are based on 18 items from DSM-IV ADHD symptomatology criteria and 8 items from the diagnostic criteria for oppositional defiant disorder. The items are scored on a four-point scale from 0–3, and the mean value of each subscale item is calculated. A single mean value of more than 1.2 is used as a possible diagnosis of ADHD [8].

### Processing and interpretation

The data collection was conducted in a room which was specially decorated and did not make the children feel uncomfortable. After a brief communication with the parents and children, they were separated by the assistants. Parents were asked to finish the SNAP-IV scale and they should not talk to their children nor guide them during the ten tasks. Then the child finished the test of the WeDA system under the guidance. First, the assistant helped the child to put on the wearable sensors on his hands, legs, waist and head and tuned the machine to ensure stable data collection. Next, the assistant told the child how to complete the game tasks until the child was able to understand and complete all the game tasks correctly. When the child started performing the game tasks, the system would capture the movement data and task results from the six wearable sensors and gathered them into a database. The child would decide the order of the tasks on its own preference.

### Statistical analysis

All the statistically analyses were performed by using SPSS 25.0 software (IBM, Armonk, NY, USA). Age and gender difference between the ADHD group and normal controls was tested using t-test and chi-square test, respectively. Calculated indicators for diagnostic validity included sensitivity, specificity, Youden index, diagnostic odds ratio, and likelihood ratio. Performance of diagnostic tests included predictive values as well as diagnostic accuracy, and comparisons of sensitivity specificity between the two groups were analysed using McNemar’s chi-squared test for paired data. In addition, the ROC curves were plotted and Z-test was performed to test whether there was a difference in the area under the curves (AUC), it is generally accepted that accuracy is high when the AUC is 0.7 to 0.9 [26].

## Results

### Subject characteristics

A total of 110 subjects were included in this study, and 55 of them were in the ADHD group and 55 were in the control group. There were no difference in the age (8.8 ± 1.76 vs 8.95 ± 1.50, *p* = 0.641) and gender ratio (Male/Female, 47/8 vs. 40/15, *p* = 0.101) between these two groups.

### Diagnostic efficacy

As shown in Table 1, the WeDA system detected 52 positives in the 55 ADHD group and 54 negatives in the 55 controls. SNAP-IV detected 42 positives in the 55 ADHD group and 44 negatives in the 55 controls. The WeDA system had a higher sensitivity (94.55% vs. 76.36%, *p* = 0.021) and specificity (98.18% vs. 80.36%, *p* = 0.002) compared to that of SNAP-IV. Furthermore, the WeDA system also had higher a Youden Index, predictive efficacy, and diagnostic accuracy than SNAP-IV (Table 2).

**Table 1:** WeDA system and SNAP-IV results of all subjects

	**WeDA System**		**SNAP-IV**	
	**Positive(n)**	**Negative(n)**	**Positive(n)**	**Negative(n)**
**ADHD group (*n* = 76)**	**52**	**3**	**42**	**13**
**CON group (*n* = 76)**	**1**	**54**	**11**	**44**
**Total (n)**	**53**	**57**	**53**	**57**

**Table 2:** The diagnostic efficacy calculation results of WeDA system and SNAP-IV

	**Specificity (%)**		**Sensitivity (%)**		**Youden index**	**PLR**		**NLR**		**Diagnostic odds ratio**	**PPV (%)**		**NPV (%)**		**Diagnostic accuracy (%)**
	**point value**	**95%CI**	**point value**	**95%CI**		**point value**	**95%CI**	**point value**	**95%CI**		**point value**	**95%CI**	**point value**	**95%CI**	
**WeDA System**	**94.55**	**83.93–98.58**	**98.18**	**89.01–99.91**	**0.93**	**52**	**7.45–362.98**	**0.06**	**0.02–0.17**	**935.99**	**98.11**	**88.62–99.90**	**94.74**	**84.45–98.63**	**96.36**
**SNAP-IV**	**76.36**	**62.67–86.34**	**80.36**	**67.16–89.33**	**0.57**	**3.89**	**2.24–6.74**	**0.29**	**0.18–0.48**	**13.22**	**79.25**	**65.5–88.70**	**77.59**	**64.40–87.08**	**78.18**

*NLR* Negative likelihood ratio, *NPV* Negative predictive value, *PLR* Positive likelihood ratio, *PPV* Positive predictive value

**Fig. 3:**
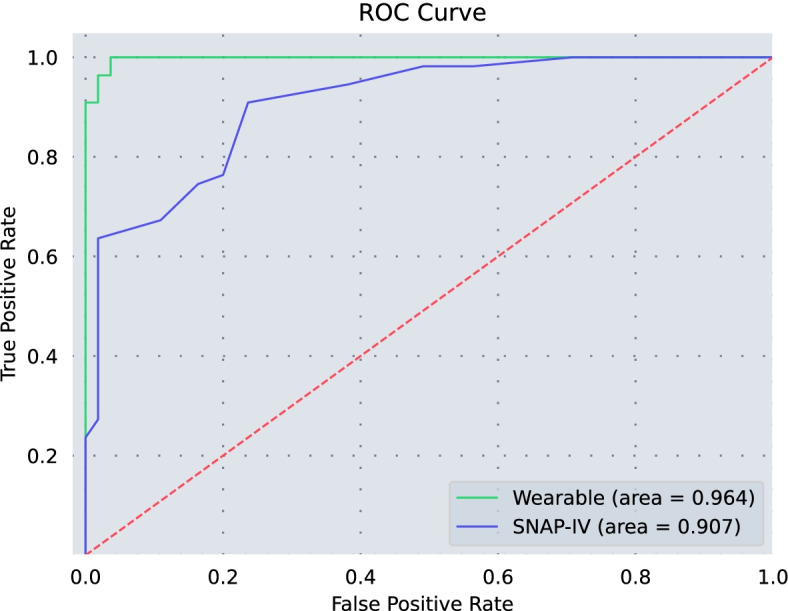
The ROC curve of WeDA system and SNAP-IV

The ROC curves of the WeDA system and SNAP-IV were presented in Fig. 3. The AUC of the WeDA system (0.964) was slightly higher than that of SNAP-IV (0.907), and the difference between them was not statistically significant (*p* = 0.068).

## Discussion

The current study found that both the WeDA system and SNAP-IV had good diagnostic value as indicated by the AUC. The sensitivity and specificity of SNAP-IV in this trial were similar to that of previous study [27]. Compared to SNAP-IV, the WeDA system showed better diagnostic accuracy in terms of sensitivity, specificity, likelihood ratio, and other indexes. The reason for this might be that the WeDA system is straightforward to use and the data are objective and less likely to be influenced by subjective factors.

Using accelerometry as an objective diagnostic indicator for children with ADHD has been studied for a long time with favorable results [17, 28, 29]. Furthermore, Niamh O’ Mahony et al. [20]. used an accelerometer together with a gyroscope to form an inertial measurement unit to distinguish the ADHD from control with an accuracy and sensitivity of up to 95%. Besides, deep learning based large amounts of motor data has been utilized for the diagnosis of ADHD. Munoz et al. [30] record the subjects’ motion for 6 school hours with accelerometers and analyzed it with Convolutional Neural Network (CNN), a deep learning model, to differentiate ADHD. The result showed an accuracy rate of 93.75%. However, using Recurrent Neural Networks, they analyzed the data that collected similarly and failed to find a significant difference between the ADHD group and the control group [18]. In the latest study, Amado-Caballero et al. placed a triaxial accelerometer on the wrist and used CNN to analyze 24-h activity data [21]. The results obtained a mean sensitivity of up to 97.62%, a specificity of 99.52%, and an AUC value of over 99%.

However, most of the previous studies have suffered from a number of shortcomings, with some studies identifying ADHD by recording motion data in a “free-living” environment being too poorly interpreted and difficult to convince. On the other hand, studies that record motion data in restricted situations do not have a good design concept and lack a theoretical basis for the task. It is also difficult to convince clinicians of the machine's diagnostic results. The rationale for this study was therefore to explore a more objective and convincing evaluation method to help clinicians make a quick, efficient and accurate diagnosis of ADHD.

Just like SNAP-IV interprets and quantifies DSM-IV, The WeDA system is also designed to cover all symptom items in the DSM-5. The ten carefully designed games include comprehensive coverage of the 18 DSM-5 diagnostic criteria of “inattention” and “Hyperactivity and impulsivity”, and are designed to incorporate doctors’ expertise. For example, when designing the grasshopper catching game, Professor Zheng Yi once said, “If a child is able to catch a grasshopper in a meadow, then he/she definitely has no ADHD. The reason for this is that catching a grasshopper requires patient observation, slow approach, holding one's breath and coordination between the hands and eyes, all characteristics that are most lacking in people with ADHD.” This is the source of the design of the grasshopper catching game. Similar ideas are used throughout the design of the game. Secondly, to assess the cognitive state of the child more comprehensively, several classical testing paradigms were used simultaneously in the design of the testing scenarios, including the Trail Making Test (TMT), Go/No-Go Paradigm, Continuous Performance Task (CPT), Stroop Effect, Wisconsin Card Sorting Test (WCST), and Stop Signal Task (SST). As a result, these more clinically relevant designs make the WeDA system more scientific. The results of the tests are more interpretative and more convincing to clinicians. Clinicians are able to use the WeDA system to clearly identify a child’s symptoms and match them to the DSM-5, providing evidence for diagnosis and improving diagnostic efficiency.

As an auxiliary diagnostic tool, the WeDA system has been designed to improve on the shortcomings of previous research, making the machine diagnostic system more scientific and more accepted among the clinician community. However, the WeDA system, as a very rudimentary prototype, has a number of shortcomings. First, The WeDA system has full coverage of the DSM-5 and incorporates multiple psychological paradigms, which has led to the design of a large number of game tasks and the possibility of some overlap between game tasks. Second, during the study, we found that subjects who completed part of the game tasks were also able to achieve a high diagnostic accuracy rate. Therefore, future study should focus on making WeDA system more simplified and personalized. Third, the WeDA system performs poorly in the classification of ADHD subtypes. The sensitivity and specificity of the ADHD subtype classification are poor. When it comes to ADHD, no single diagnosis or treatment can be applied to everyone, as each patient will have different symptoms and the corresponding treatment will be different. Hence, more efforts are needed to have a complementary diagnostic technique in order to make an accurate diagnosis for ADHD subtypes.

## Conclusion

The WeDA system has excellent clinical diagnostic performance. Compared with SNAP-IV, the WeDA system has superior sensitivity, specificity, and accuracy in all aspects. The advantages of the WeDA system in terms of diagnostic objectivity, scientific design and ease of operation make it a promising system for widespread use in clinical practice.

## Data Availability

The data that support the findings of this study are available on request from the corresponding author. The data are not publicly available due to privacy or ethical restrictions.
